# Red - Take a Closer Look

**DOI:** 10.1371/journal.pone.0108111

**Published:** 2014-09-25

**Authors:** Vanessa L. Buechner, Markus A. Maier, Stephanie Lichtenfeld, Sascha Schwarz

**Affiliations:** 1 Ludwig-Maximilians-Universität München (LMU Munich), Munich, Germany; 2 University of Wuppertal, Wuppertal, Germany; Monash University, Australia

## Abstract

Color research has shown that red is associated with avoidance of threat (e.g., failure) or approach of reward (e.g., mating) depending on the context in which it is perceived. In the present study we explored one central cognitive process that might be involved in the context dependency of red associations. According to our theory, red is supposed to highlight the relevance (importance) of a goal-related stimulus and correspondingly intensifies the perceivers’ attentional reaction to it. Angry and happy human compared to non-human facial expressions were used as goal-relevant stimuli. The data indicate that the color red leads to enhanced attentional engagement to angry and happy human facial expressions (compared to neutral ones) - the use of non-human facial expressions does not bias attention. The results are discussed with regard to the idea that red induced attentional biases might explain the red-context effects on motivation.

## Introduction

The color-in-context theory [1]–[3], a recent model of color and psychological functioning, posits that color automatically triggers evaluative processes, which in turn influence psychological functioning (see also [4], [5]). These color effects are supposed to be grounded in a situative context [2], [3]. Recent research on the color red has shown that red carries the meaning of ‘danger’, ‘threat’, and ‘caution’ [6], triggers avoidance motivation and aggressive responses, and undermines performance in various forms of achievement contexts [7]–[10]. In the context of affiliation, however, red has been shown to be associated with the meaning of ‘attractiveness’, ‘sex’, and ‘romance’ and thus to activate approach motivation [11]–[15]. These context-dependent effects of the color red on motivation have also been confirmed by Meier and colleagues [16] who used a within study experimental manipulation of context. Even though the contextual framing of the meaning of the color red seems to be empirically well established, up to date, nothing is known about the exact nature of the underlying psychological mechanisms. Buechner, Maier, Lichtenfeld, and Elliot (unpublished data) proposed that red is a signal of relevance which carries the message that a present stimulus is important and worthy of attention [17]. This attentional bias then emphasizes the stimulus’ motivational message and thus increases the perceivers’ existing response tendencies. In other words, the red induced attentional bias is supposed to be an antecedent/prerequisite of context-specific red effects. This attentional biasing effect of red was tested in the present research.

### Red and goal-relevance

Plenty of studies have documented effects of the color red on perceptual processes of social stimuli across different research areas. According to research investigating the influence of facial makeup on women’s attractiveness, red seems to play a fundamental role in the perception of faces (e.g., [18]–[25]). Additional research focused on the influence of the color red and other colors on perceptions of sex and ethnicity [26], [27], age [28]–[30], identity [31], [32], health [33], [34], and attractiveness [11], [12], [15], [29], [30], [35], [36]. In temporally and geographically diverse cultures reddish cheeks and lips enhance apparent perceptions of health and attractiveness, particularly in female faces [37]–[39], as these reflect higher estrogen levels [40], [41]. In addition, human perceptions of sexual attraction are accompanied by red skin flushing that spreads from the chest to the face [42]. Also, increased red coloration of the chest or genitals of some primates signals fertility and sexual receptivity [43]–[45] and thus facilitates approach behavior in potential mates [46], [47].

However, increased blood flow to the face or chest is also associated with anger toward and dominance over potential opponents in both humans and other primate species [48]–[54], which is accompanied by avoidance behavior within the observer [55], [56]. Specifically, the experience of anger increases red flushing of the skin in both men and women [57], [58], and these red faces in turn are perceived as more angry and dominant [52]. Also, Young and colleagues [59] found that the color red enhances the perception and identification of angry faces. Some findings even indicate that the color red seems to highlight the importance of stimuli. Changizi and colleagues [51] argue, that red signals have driven the primates’ red-green color system evolution.

In sum, red skin coloration has been shown to have effects on two different motivational tendencies namely approach as in the case of affiliation and avoidance as in the case of danger, by either accentuating engageable features of others or highlighting anger and dominance of potential opponents. Even though, research indicates that red seems to facilitate perception and identification of these features [59], it remains open whether these red effects are related to basic attentional mechanisms.

### Goal-relevance and Attention

During the last two decades a growing number of studies have investigated the relationship between motivational states and attention (e.g., [60]). The posterior attentional system [61] has been shown to automatically orient attention from one particular stimulus to another. Interestingly, this system is supposed to be guided by motivational states that enable motivationally relevant stimuli to capture attention [62]. McArthur and Baron [63] suggest that individuals selectively process key features that are relevant to the satisfaction of their motives. Thus, motive activation is presumed to influence cognitive processes such as attention (e.g., [64]). Specifically, motivational states promote automatic attentional biases due to the inefficiency of disengaging attention (i.e., enhanced attentional adhesion) from goal-relevant stimuli. Theories of sexual selection, for instance, propose an attention bias toward physically attractive members of the opposite sex (e.g., [65]). This bias arises because physical attractiveness serves as a potential sign of health, fertility (e.g., [66]), and high genetic fitness, which in turn increases the likelihood of having genetically fit and healthy offspring [67]–[70]. In addition, self-protective motives, as well as trait and state anxiety, have been shown to enhance attentional vigilance toward threatening stimuli [71]–[73].

Taken together, research on motivation and attention clearly indicates that motivational states are often eliciting an intensified attentional processing mode. Apparently, a perceiver’s motivational goal has some potential to manifest itself in different forms of biased attention toward stimuli that might fit the goal.

### The Present Research

The color red seems to be a cue of motive relevance and motivationally-relevant stimuli seem to attract attention. Hence, in an attempt to explain context dependent effects of the color red, Buechner et al. (unpublished data) recently suggested that the color red intensifies attention to goal-relevant stimuli. Human beings, already early in their life, seem to be predisposed to identify the emotional content of human faces for the purpose of reacting accordingly [74]. Thus, we decided to focus on this omnipresent class of goal relevant stimuli, that is, humans’ emotional expressions (happy and angry). We hypothesize that red increases the targets’ power to capture the perceiver’s attention and thus leads to enhanced attentional adhesion (i.e., reduced disengagement). A paradigm designed to identify variations in attentional adhesion is the so-called “dot probe task”, which measures participants’ efficiency in shifting attention away from a particular stimulus [72], [75]. In the study presented herein, we use this dot probe task to measure increased attentional adhesion of red primed angry and happy human facial expressions in comparison to various kinds of control conditions.

## Method

### Ethics Statement

The research reported herein was conducted at the LMU Munich and was approved by the ethics committee of the Department of Psychology, LMU Munich, in accordance with the ethical standards expressed in the Declaration of Helsinki. All participants gave verbal informed consent and were thoroughly debriefed. Verbal consent was considered to be sufficient, since it was ensured that data were stored and analyzed anonymously. The individuals’ verbal consent was obtained after reading the instruction to the experiments. The experimenter asked for the participant’s consent and emphasized that they will receive their credit also if they decided not to participate in this study. Participants were also told that they could stop and leave the experiment at any point of time. This consent procedure has been approved by the ethics committee.

### Participants

159 undergraduates (148 women, *mean age* = 23.28 years, *SD* = 6.59) participated in this study for course credit. Participation was restricted to individuals who did not have a color deficiency.

### Design and procedure

Participants were randomly assigned to a between-subjects target face condition (human vs. non-human) and were run individually by an experimenter blind to participants’ condition and the experimental hypothesis. They performed a modified visual dot probe task [60] whose objective is to measure attentional adhesion to goal relevant stimuli. This task has extensively been used by Maner and colleagues in a series of studies testing attentional processing of visually presented goal relevant material. Specifically, they found that successful goal activation slowed down attentional distraction for goal relevant objects confirming the link between motivational goal orientation and attention [60].

**Figure 1:**
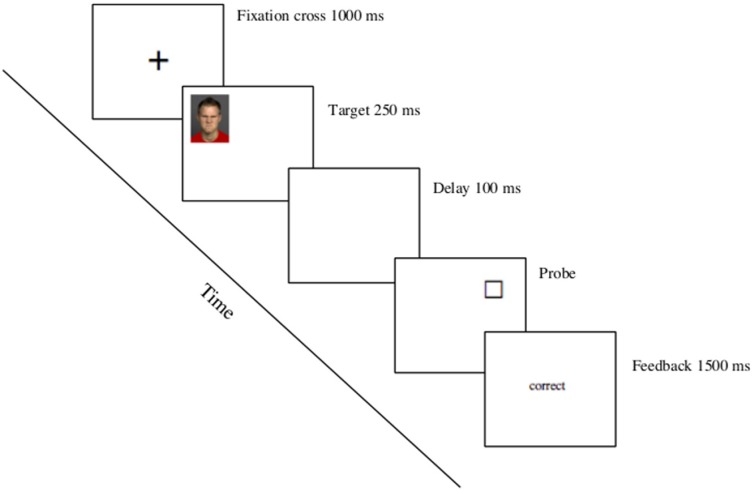
Example of the procedure.

The dot probe task was created using the E-Prime stimulus presentation program (Psychology Software Tools, Pittsburgh, PA) and the procedure for each trial was as follows: First, a fixation cross (X) appeared in the middle of the computer screen for 1.000 ms. Concurrent with the disappearance of the fixation cross, a target face was displayed for 250 ms in one out of four quadrants of the screen (i.e., upper right, upper left, lower left, or lower right). After an interstimulus interval of 100 ms, a categorization object (circle or square) appeared at either the same position as the target face (filler trials) or in a different quadrant (attentional shift trials, that are the trials of interest, see Figure 1 for an example of the procedure). Hence, on attentional shift trials, participants need to shift their attention away from the location of the target face to a different position on the screen. Importantly, we used exposure durations of 250 ms, instead of 500 ms (e.g., [60]) to replicate the pattern of early vigilance followed by later avoidance reported by Cooper and Langton [76]. Each block contained 3 filler trials and 9 attentional shift trials. Shift and filler trials were randomly presented. Participants were told to categorize the object as a circle or square by pressing the A or K key, respectively, as quickly and accurately as possible. The response latency between the appearance of the object and the participant’s key response is the measure of attentional adhesion: While reduced response times indicate that it took less time to shift the attention away from the target face, larger response times indicate enhanced attentional adhesion. In line with Maner and colleagues [60] only response latencies on shift trials were analyzed. Once the participant categorized the object, feedback was provided for 1.500 ms, followed by a 2.000 ms break before the next trial. Participants were given 16 practice trials to familiarize themselves with the procedure, followed by four blocks of 12 experimental trials in a random order within each block. The position of the target face and position of the object type (circle or square) were randomized across trials, so that each appeared in either location with equal probability for each type of face.

**Figure 2:**
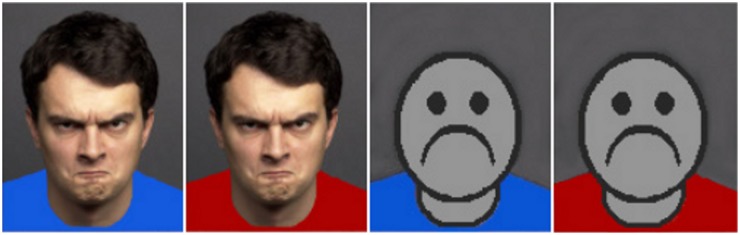
Example of the type of stimuli used.

The stimuli used in the present study consisted of 48 target pictures (4 human male faces were selected from FACES, a validated database of facial expressions by the Max Planck Institute for Human Development [77]) and 4 non-human faces (i.e., neutral abstract faces, frownies, and smileys); each face with an angry, happy, and neutral facial expression, respectively, and each of these 24 faces with a red and blue T-shirt, respectively, rendering 48 pictures in total), revealing a color 2 [red, blue]×emotion expression 3 [angry, happy, neutral]×2 target face [human, non-human] mixed design ANOVA with color and emotion expression as within-subjects factors and target face as a between-subjects factor. By using Adobe Photoshop, the T-Shirts were colorized in red or blue. A GretagMacBeth Eye-One Pro spectrophotometer was used to select red and blue colors that were equivalent on lightness and chroma, revealing parameters for red LCh (39, 77, 40) and for blue LCh (39, 77, 282). This contrast allows a highly controlled test of the effect of hue holding the other two color properties constant. We included both, human and non-human facial stimuli to test for the role of the color red on stimuli that are specifically socially relevant (see Figure 2 for an example of the type of stimuli used). We expected red effects on attentional adhesion rather for emotional human faces than for non-emotional and non-human facial stimuli, given the importance of human faces for social communication reported in the literature (e.g., [78], [79]). Emotional human facial stimuli specifically function to non-verbally communicate motive-relevant information to the perceiver (see [80]–[83] for the goal-relevance of human faces). Displaying such a signified message has been shown to enhance the perceivers’ attention [84]–[86]. With regard to the color red, as a signal of relevance, we propose that it should additionally intensify the motive-relevance of valenced faces and thus causing increased attentional adhesion toward them compared to control faces. Pictures used reveal a high intensity of the prototypical facial expression and are standardized regarding the size and distances of the head in the picture to the image borders (there were slight deviations due to differences in head sizes, neck lengths, and hairstyle, see [77]). To ensure the same brightness, Ebner and colleagues [77] matched all pictures to a predetermined standardized matrix image by using Adobe Photoshop CS. All face images were presented at the same size on a color-calibrated screen and participants sat approximately 50 cm away from the screen in a darkened room.

After finishing the dot probe task, participants answered some demographical questions and were debriefed and dismissed. No participant reported awareness of the purpose of the task.

### Measures

Participants’ reaction time (in milliseconds) with which they responded on attentional shift trials served as the dependent variable. Separate measures of attentional adhesion to red and blue human and non-human faces with an angry, happy, and neutral facial expression were calculated. According to Maner and colleagues [60], participants with highly aberrant data (response times greater than 3.0 standard deviations above or below the sample mean) and trials with incorrect categorizations should be removed from analysis. Notably, none of the participants fulfilled these criteria, so neither participants nor trials were removed in the present study.

## Results

First, a 2×3×2 mixed design ANOVA with color (red vs. blue) and emotion expression (angry vs. happy vs. neutral) as within-subjects factors and target face (human vs. non-human) as a between-subjects factor was conducted on attentional bias, to compare attentional adhesion to human and non-human targets with an angry, happy, and neutral facial expression in the red and blue color condition. Our prediction was that attentional adhesion should be found in the red condition for valenced (i.e. angry and happy) human faces compared to neutral ones. No color×emotion expression effects were expected for the non-human stimuli.

The analyses revealed the predicted three-way interaction between color, emotion expression, and target face, *F*(2,156) = 3.07, *p* = .048, *η* ^2^_p_ = .02. No significant main effect of emotion expression (*F*<1) nor color (*F*<1) was found. Only the main effect of target face was significant, *F*(1,157) = 13.19, *p*<.01, *η* ^2^_p_ = .08, with non-human faces being processed less quickly (*M* = 501.07 ms, *SD* = 85.29) relative to human stimuli (*M* = 437.83 ms, *SD* = 130.00). Within the human condition, the analyses revealed the predicted two-way interaction between color and valence, *F*(2,77) = 2.46, *p* = .09, *η* ^2^_p_ = .06. No significant main effect of valence (*F* = 2.00, *p* = .14) nor color (*F* = 1.18, *p* = .28) was found. Within the non-human condition, the analyses revealed no two-way interaction between color and valence, *F* = .70, *p* = .50, as well as no significant main effect of valence (*F* = .12, *p* = .89) or color (*F* = .02, *p* = .88).

Next, we analyzed the data separately for the human and the non-human faces conditions. Abelson and Prentice [87] as well as Rosenthal and Rosnow [88] suggested the use of a set of a priori contrasts for the analysis of effects obtained in an ANOVA. Following this, we first tested the existence of a valence effect within the human faces condition. A Helmert contrast comparing neutral vs. valenced (i.e., angry and happy) faces revealed a marginally significant effect, *F*(1,78) = 3.44, *p* = .067, *η* ^2^_p_ = .04, indicating that angry (*M* = 442.69 ms, *SD* = 131.17) and happy faces (*M* = 438.59 ms, *SD* = 133.72) produced more attentional adhesion than neutral ones (*M* = 432.20 ms, *SD* = 134.74). The same Helmert contrast performed within the non-human faces condition revealed no valence effect on attentional adhesion, *F*<1.

**Figure 3:**
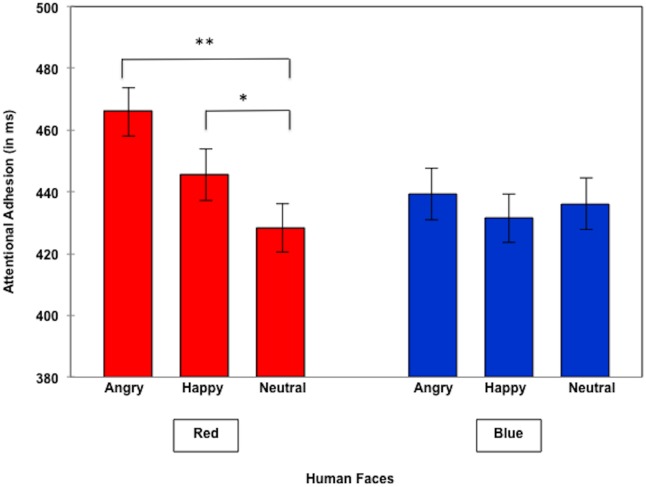
Attentional adhesion to human faces.

Next we tested our hypothesis that red increases attentional adhesion for valenced compared to neutral human faces, but not for non-human ones. For human faces within the red condition the corresponding Helmert contrast (neutral vs. valenced faces) was significant, *F*(2,156) = 3.44, *p* = .034, *η* ^2^_p_ = .04. Additionally performed planned contrasts revealed increased attentional adhesion for angry (*M* = 446.19 ms, *SD* = 128.85) versus neutral faces (*M* = 428.47 ms, *SD* = 131.92), *t*(78) = 2.64, *p* = .01, *d* = .37 (see Figure 3). A similar effect was found for happy (*M* = 445.59 ms, *SD* = 140.72) versus neutral faces (*M* = 428.47 ms, *SD* = 131.92), *t*(78) = 2.19, *p* = .03, *d* = .19 (see Figure 3). No significant difference was found for angry versus happy facial expressions, *t*<1. In the blue baseline condition, no significant differences were found, all *t*s<1 (see Figure 3).

In addition another set of planned contrast was performed. Color effects (red vs. blue) were tested separately within the valenced (angry and happy) and the neutral facial target condition. The analysis performed with valenced stimuli yielded a marginally significant color effect, revealing increased attentional adhesion for red (*M* = 445.89 ms, *SD* = 129.69) versus blue valenced faces (*M* = 435.39 ms, *SD* = 134.21), *t*(78) = 1.91, *p* = .06, *d* = .21. No significant effect was obtained for neutral faces (red, *M* = 428.47 ms, *SD* = 131.92, versus blue neutral faces, *M* = 435.94 ms, *SD* = 142.82, all *t*s<−1.22). An additional set of color contrasts performed separately for angry and happy human faces revealed no significant color effect for angry faces (red, *M* = 446.19 ms, *SD* = 128.85, versus blue angry faces, *M* = 428.47 ms, *SD* = 131.92, *t*<1). However, there was a color trend for happy faces, yielding increased attentional adhesion for red (*M* = 445.59 ms, *SD* = 140.72) versus blue happy faces (*M* = 431.60 ms, *SD* = 135.18), *t*(78) = 1.83, *p* = .07, *d* = .21. It seems that the color effect on attentional adhesion within the group of valenced human faces was primarily driven by happy facial expressions. To test whether the red-happy effect was driven by the somewhat low red-neutral baseline, as one of the reviewers suggested, we checked whether red-neutral was significantly different from any of the blue conditions (all the other possible baseline conditions). All *t* scores obtained were less than 1.6, all *p*s>.10, indicating that the red neutral baseline was not an outlier. We also performed an analysis red-happy vs. blue-angry/blue-neutral, and found that it was not significant, all ts<1.11. This latter finding - but only if regarded in isolation- causes some doubt in the validity of the red-neutral score as a baseline when comparing it with the red-happy mean score.

**Figure 4:**
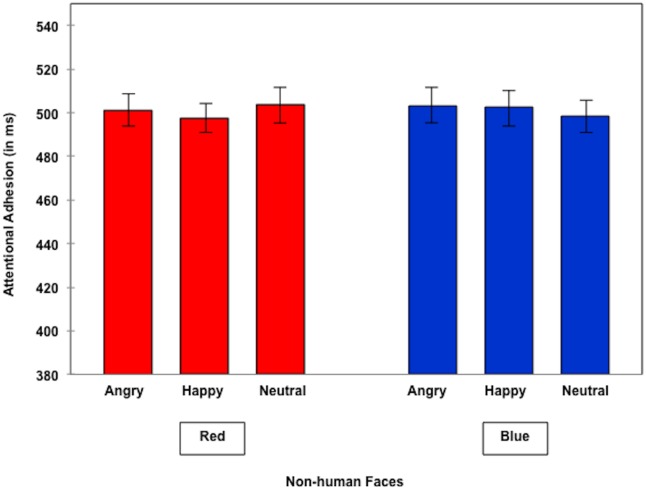
Attentional adhesion to non-human faces.

The same analyses performed within the non-human faces condition revealed no significant effects. No significant differences neither with the Helmert contrast (neutral vs. valenced faces, *F*<1) nor with any of the additionally performed contrasts neither in the red nor in the blue condition were found, all *t*s<1 (see Figure 4). Also, none of the color contrasts for any of the emotion expression (valenced or neutral) conditions was significant, all *t*s<1.004.

Taken together, in line with our predictions red seems to increase attentional adhesion for valenced human faces compared to neutral ones and compared to a blue control color condition. No red effect was found for non-human stimuli.

Supplementary analyses. Since only male target pictures were presented we explored the potential moderating role of participants’ gender on the attentional adhesion effect reported above. From a theoretical point of view, such moderating effects of gender have not originally been expected: Although males might differ from females with regard to the exact motive under which a valenced facial stimulus is perceived, for instance a smiling man might induce approach tendencies in female perceivers (due to being a potential mating partner) and avoidance tendencies in male perceivers (due to being a potential competitor), nevertheless, in both instances a motive should always increase attentional adhesion regardless of its quality. As described in the method section, a disproportionally high amount of female individuals (148 out of 159) participated in this study. Thus any interaction effects with gender could not be tested. As an alternative we calculated the effect size for the three-way interaction for females only. In this way gender-specific motives possibly being present in the perceiver while watching the pictures were kept constant. An ANOVA conducted in the female sample with all the factors described above again yielded a significant three-way interaction between color, emotion expression, and target face, *F*(2,146) = 3.81, *p* = .02, *η* ^2^_p_ = .025. Importantly, the effect size obtained with female participants does not differ from the effects size obtained with the whole sample (*η* ^2^_p_ = .02). In addition, an ANCOVA with gender treated as covariate also yielded the same effect size for the three-way interaction as reported above, *F*(2,152) = 3.80, *p* = .02, *η* ^2^_p_ = .024, and thus further supports that gender effects are negligible in our study.

## Discussion

Prior research has defined context in terms of domain of behavior (i.e., the achievement and the affiliation domain; [3] for a review) and results showed that the influence of color on psychological functioning varies as a function of these. However, so far the exploration of the underlying *psychological mechanisms* was neglected. In a recent attempt to shed some light on these mechanisms, Buechner et al. (unpublished data) suggested that red primarily functions as a signal of increased relevance and that it intensifies attentional processing of goal-related stimuli. In the study presented here, human faces displaying different kind of emotional states served as experimental stimuli (see [80]–[83] for the goal-relevance of human faces). We examined the combined influence of color and emotion expression on increased attentional adhesion to human and non-human targets. We anticipated increased attentional adhesion in the red condition for angry and happy human faces compared to neutral ones. In addition, we anticipated no such effects in either the blue human or the non-human conditions. As predicted, within the red condition, increased attentional adhesion for angry and happy versus neutral human faces was found and no such effects emerged within the blue condition. Further, for the non-human targets no color×emotion expression effects were obtained confirming the idea that red only increases attentional adhesion for socially relevant targets (i.e. human faces). In comparison to non-human targets, human targets are more meaningful for an individual, as they convey crucial information with regard to an observer’s social goals such as approaching happy and thus friendly individuals to engage with them and avoiding potentially threating ones as in the case of anger. According to our interpretation, red increases the personal relevance of such existing affective, goal-related information and thus to a higher degree attracts an individual’s attention.

Although not tested, our theoretical model also postulates that in a second step attentional adhesion serves as an intensifier of ongoing motivational tendencies (Buechner et al. demonstrated that red was indeed an intensifier of a motivational tendency without referring to attention [unpublished data]). This idea of attention being the moderator of motivation bears some parallels with existing theories and research on attention and motivation. For instance, Gable and Harmon-Jones [89] could demonstrate that manipulations of attentional focus toward affective stimuli also increased electrophysiological emotional responses to them. According to their research, attentional processes (in this case focus of attention) and experienced motivational intensity were closely related to and causally affected by each other (see [90]). Taken together, in the present study, context is represented by valenced faces, as emotional faces are known to represent goal- relevance [80]–[83] and thus ultimately elicit approach and avoidance tendencies. In addition, according to our theory, in an early processing step any motivational context (approach and avoidance) should produce increased attentional adhesion when red is involved. The context-specific differential effects of the color red on behaviors (as described in the previous color literature) should then emerge in a later stage of processing. Specifically, red in avoidance related contexts (e.g. achievement or threat) should produce avoidance reactions and fear whereas red in approach related domains should elicit approach behaviors and hope. In other words, attentional adhesion is supposed to be the initial indicator of relevance in order to support these contextually framed behavioral strategies. We are aware of the fact that the present study only constitutes a first step of testing the idea that context effects of the color red on motivation are mediated by attentional adhesion mechanisms and future studies should explore this mediational role. However, we think that identifying attention as a potential promising candidate is an important first step. In our view we successfully managed this initial research goal with a highly powerful sample and carefully designed control conditions.

We also think that the color red might not be the only factor that serves the purpose of increasing the relevance and motivational power of a stimulus. Additional potential candidates amongst others may be target position on the screen, display duration, or picture size. These and other factors as well as their effects on contextual variations of motivational goals should also be explored in future research. Nevertheless, red played a central role in our research on “motivation in context” as it is a stimulus that naturally occurs and can easily vary on human faces (e.g., [18]–[25]).
